# The miR‐192‐EGR1/HOXB9 Loop Regulates Glioma Cell Stemness and Malignant Phenotypes by Promoting Their Mesenchymal Transition

**DOI:** 10.1111/jcmm.70842

**Published:** 2025-09-11

**Authors:** Guo‐Wei Li, Yan‐Ping Jin, Min‐Feng Sheng

**Affiliations:** ^1^ School of Rehabilitation Science Nanjing Normal University of Special Education Nanjing Jiangsu China; ^2^ School of Nursing Jiangsu Health Vocational College Nanjing Jiangsu China; ^3^ Department of Neurosurgery The Second Affiliated Hospital of Soochow University Suzhou Jiangsu China

**Keywords:** GSCs, loop, malignant phenotype, mesenchymal transformation, stemness

## Abstract

To clarify the regulatory effects of miR‐192 on the malignant phenotypes of glioma cells. We used PCR, WB and immunofluorescence to detect regulatory factors in glioma samples. Then, we chose lentiviral plasmid transfection to construct cell models. We used CCK‐8 and colony formation to evaluate the proliferation ability of these cells and used Transwell/scratch tests to evaluate their invasion ability. CD133‐expressing GSCs were observed under a microscope, and their stemness properties were evaluated. We constructed a tumour‐bearing model via subcutaneous inoculation. Tumour growth curves and tumour weights were determined subsequently. The proteins involved in the miR‐192‐EGR1/HOXB9 loop were evaluated via IHC staining. MiR‐192 was significantly reduced in glioma samples, and this factor downregulated EGR1 and HOXB9 via targeted binding, thus forming a semi‐open loop. Moreover, the proliferation, invasion and migration of glioma cells overexpressing miR‐192 were significantly decreased. These malignant phenotypes were abrogated completely with EGR1 or HOXB9 overexpression. Similarly, these changes were essentially consistent with MT marker expression and the stem‐like properties in glioma cells. Meanwhile, miR‐192 inhibits the tumorigenesis of glioma cells via the EGR1‐HOXB9 loop. MiR‐192 could inhibit MT in glioma cells through the EGR1‐HOXB9 loop. Thus, it reduces their stemness and abrogates their malignant phenotypes.

**Abbreviations**
CSC: cancer stem cell
EGR1: early growth response protein 1
GSCs: glioma stem cells
HOXB9: homeobox genes 9
MT: mesenchymal transition
TCGA: The Cancer Genome Atlas

## 1 Background

Glioma is a common primary intracranial tumour (accounting for 80% of malignant tumours) with strong aggressiveness and high recurrence, morbidity and mortality rates. At present, the primary treatment method is still surgical resection; however, high‐grade cases (III–IV) often show invasive growth, with no clear demarcation between tumour tissues and the surrounding tissues. Some lesions are located deep in the brain within important functional areas and are difficult to completely remove. Therefore, residual lesions often exist, affecting patients' postoperative recovery and putting them at risk of postoperative recurrence [1, 2]. Exploring the molecular mechanisms underlying the invasiveness of glioma cells and adopting targeted measures may aid in the successful treatment of glioma. However, the molecular mechanisms have not yet been fully elucidated.

Recent studies have shown that the invasive growth of glioma cells into surrounding tissues may be closely related to the pathophysiological process of mesenchymal transition (MT). In tumour cells undergoing MT, the tight junctions between cells are usually lost, resulting in the cells acquiring mesenchymal characteristics such as high invasiveness, which is a key initiating step in the invasion and metastasis of malignant tumours [3, 4]. Some scholars have reported that MT may play key roles in the malignant progression of cancers and that MT is closely associated with the development of cancer stem cells (CSCs) [5, 6]. In recent years, researchers have successfully isolated and identified glioma stem cells (GSCs) from glioma samples; nevertheless, whether these cells undergo MT and whether this pathophysiological process is involved in glioma cell invasiveness remain controversial.

Previous studies have shown that miR‐192 can arrest the cell cycle in the G1/G2 phase and that its expression is significantly downregulated in tumour tissues such as colon and endometrial tumour tissues. Therefore, this regulatory factor is considered a tumour suppressor [7]. Zhang Tao confirmed that the expression of miR‐192 in glioma cells is significantly reduced and that this factor can inhibit the proliferation and migration of glioma cells. Moreover, the apoptosis rate of U251 cells obviously increased with the upregulation of miR‐192. EGR1 (Early Growth Response Protein 1) is a member of the early growth response protein family and has been identified as a downstream regulatory molecule of growth factors, hormones, neurotransmitters and metabolites in multiple physiological processes. The expression of EGR1 in prostate cancer tissues is greater than that in the surrounding tissues; moreover, its expression level is positively correlated with the degree of tumour malignancy. Similar clinical associations were found in gastric cancers [8, 9]. However, the role of EGR1 in glioma has rarely been reported in the literature. HOXB9 (Homeobox Genes 9) belongs to the HOX family and is usually abnormally expressed in breast and lung cancer. This factor plays pivotal roles in promoting growth, invasion, apoptosis and other processes in tumour cells [10]. Sherry Y's team confirmed that miR‐192, EGR1 and HOXB9 might be regulated by the same upstream factors and thus participate in tumour angiogenesis and invasion [11]. However, until now, no relevant studies have reported the expression of pathway members in glioma cells or the impact of the pathway on the malignant phenotype of glioma.

On the basis of these previous results and our bioinformatics analysis, we proposed that the miR‐192/EGR1‐HOXB9 loop could mediate MT in glioma cells and regulate their stemness, thereby participating in the regulation of glioma cell malignant phenotypes. In this study, we conducted in vitro cell experiments and in vivo studies to evaluate tumour cell phenotype, tumour histology and molecular changes to verify these findings. This study aimed to elucidate the molecular regulatory mechanisms involved in the malignant phenotypes of glioma cells, thereby providing a theoretical basis for the clinical treatment of high‐grade glioma patients.

## 2 Materials and Methods

### 2.1 Cell Culture

U87 and U251 cells were thawed and resuspended in DMEM‐H (containing 10% foetal calf serum + 1% penicillin–streptomycin), after which they were cultured at 37°C and 5% CO_2_. The cells were passaged when 80%–90% of the cells were attached to the wall, and the cells in the logarithmic growth phase, which occurred after passage 3, were used for subsequent experiments.

### 2.2 Lentiviral Transfection

Lentiviruses carrying plasmids were introduced into 293 T cells via the calcium phosphate precipitation method and concentrated with a Lenti‐X concentrator (Takara). Glioma cells (U87 and U251) were cultured in coated 6‐well plates. When the cell density reached 80%, Lipofectamine 2000 was used as the transfection reagent, and then OE RNA/NC and SH RNA/NC were transfected into the glioma cells. After the medium was changed, the glioma cells were cultured at 37°C and 5% CO_2_ for 48 h, and the cell transfection efficiency was observed under a fluorescence microscope.

### 2.3 CCK‐8 Assay

The transfected U87 and U251 cells were seeded in a 96‐well plate at a density of 1 × 10^4^ cells/well and cultured at 37°C and 5% CO_2_. Each treatment group had three wells. Afterwards, we added 10 μL of CCK‐8 reagent solution to each well and mixed it evenly after 24, 48 or 72 h of culture. The reagent was incubated with the cells for another 4 h, after which a fully automatic enzyme marker was used to detect the optical density (OD) of the cells in each well at 450 nm.

### 2.4 Transwell Assay

The invasive ability of glioma cells was evaluated via a permeability support assay (polycarbonate membrane, Corning) using a 24‐well Transwell system. U87 and U251 cells (1 × 10^3^) suspended in serum‐free DMEM were added to the upper chamber, and 10% FBS DMEM was added to the lower chamber. After 10 h, paraformaldehyde was added for fixation, and crystal violet was added for staining. Then, we placed the migrated cells under a microscope for imaging. Finally, we selected these images to measure the crystal violet staining intensity (six fields for each group).

### 2.5 RT‐PCR

RT‐PCR was used to measure the expression of miR‐192 (5′–3′), and total RNA was isolated using a mirVana miRNA isolation kit (AM1561; Ambion Company). The first strand was synthesised using a cDNA synthesis kit, and SYBR Green I was used in the PCR detection system. U6 (5′–3′) was used as an internal reference gene for data normalisation, and the $2^{-∆∆C_{t}}$ method was used to calculate the relative expression of target RNAs.

### 2.6 Western Blot (WB) Analysis

The primary antibody was added to the membrane and incubated overnight on a shaking table at 4°C. The primary antibodies used and their dilutions were as follows: anti‐EGR1 antibody (1:5000), anti‐HOXB9 antibody (1:10,000) and anti‐GAPDH antibody (1:20,000). After 12 h, the membrane was washed with PBS 3 times (10 min). The corresponding secondary antibodies were incubated for 1 h (1:2000), and then the luminescent solution was mixed 1:1 with solution A/B and then added to the membrane for protein band development. Finally, we used ImageLab 4.2 for grayscale scanning.

### 2.7 Dual‐Luciferase Reporter Assay

To explore the binding sites between miR‐192, EGR1 and HOXB9, we generated EGR1/HOXB9 3′ UTR‐WT (containing the EGR1/HOXB9 3′ UTR fragment) and EGR1/HOXB9 3′ UTR‐MUT (containing the EGR1/HOXB9 3′ UTR fragment mutant) luciferase reporter vectors. After the cells were cotransfected with the miR‐192 mimic or mimic NC via Lipofectamine 2000 for 24 h, their luciferase activity was measured according to the instructions of the kit used in this assay.

### 2.8 Cellular Immunofluorescence

Cells on slides were washed twice in preheated PBS (3 min), fixed in 4% paraformaldehyde for 30 min and washed in PBS three times (10 min). Afterwards, the slides were incubated in 10% goat serum + 0.1% Triton at room temperature for 30 min. Then, we removed the blocking solution, added diluted primary antibody solutions, and incubated the samples in an incubator at 4°C overnight. Next, the slides were washed in PBS three times (10 min per wash), and diluted fluorescent secondary antibodies were added. Finally, we incubated these samples in an incubator at 20°C–37°C for 24 h. We washed the slides with PBS three times (10 min per wash), and DAPI was added to the slides and incubated in the dark for 5 min to stain their nuclei. The slides were washed with PBS for a final time. The slides were dried and sealed with an antifluorescence quencher. Finally, these slides were observed, and images were collected under a fluorescence microscope.

### 2.9 Colony Formation Assay

To determine the proliferation ability of glioma cells (U87 and U251) in the NC and experimental groups, 2 × 10^3^ cells from each group were selected and inoculated in six‐well plates and cultured until obvious colonies formed. These samples were stained with crystal violet (Beyotime Biotechnology), and the total number of clones in each well was counted and compared. Each experiment was repeated three times.

### 2.10 Scratch Test

U87 cells were grown in six‐well plates and transfected with plasmids for 72 h. The media was then replaced with serum‐free DMEM, and the cells were cultured for another 12 h. All the cells were subjected to three consecutive washes with PBS, and a scratch was made in the centre of the cell monolayer with a 200 μL pipette tip. After incubation for 24 h, these cells were evaluated under a microscope, and all the assays were repeated three times.

### 2.11 Isolation, Propagation and Immunofluorescence of GSCs

U87 and U251 cells were cultured in DMEM/F12 medium (10% foetal bovine serum + 1% penicillin streptomycin) for 24 h in humidified incubators at 37°C and 5% CO_2_. We subsequently replaced the medium with serum‐free neural stem cell DMEM/F12 medium (containing 1% penicillin streptomycin, 40 μg/L HEGF, 40 μg/L HFGF and B‐27 supplement at a 2% volume fraction).

GSCs with self‐renewal ability can form tumour spheres, and we separated these cells and digested them to create single‐cell suspensions. They were seeded into 6‐well plates at a high density to increase the number of GSCs. These stem cells were observed to form spheres under a microscope. After these GSCs were extracted and fixed in 4% paraformaldehyde for 30 min, they were permeabilised with Triton X‐100 for 15 min, blocked with 5% bovine serum albumin for 2 h and incubated with a CD133 primary antibody (1:400) at 4°C overnight. The cells were then incubated with the fluorescent secondary antibody at room temperature in the dark for 1 h, after which they were stained with DAPI for 10 min. Finally, the expression of the stem cell marker CD133 was detected via fluorescence microscopy.

### 2.12 Establishment of Subcutaneous Tumour Mouse Model

We selected 12 BALB/c nude mice (male, 4–6 w, 18–20 g) offered by Charles River and placed them in conditions with 20°C–26°C, 40%–70% humidity and 12 h light/12 h dark. Continuous ventilation/filtration and regular food/water were provided. We selected glioma cells (U87 and U251) in the logarithmic growth phase, adjusted the density to 1 × 10^4^/L and inoculated them subcutaneously into the left axilla of nude mice in a volume of 100 μL of cell suspension. After round tumour nodules appeared, mice were randomly divided into a control group (NC group) and an experimental group (OE miR‐192 group), with three mice in each group.

The body weights of the mice were measured on days 0–30, while the volumes of the subcutaneously transplanted tumours were evaluated (volume = 1/2 × L × W × W) on Days 6, 8, 10, 12, 14, 16, 18, 20, 22, 24, 26 and 28; growth and tumour volume curves were drawn according to these data. After the tumour samples were dissected and weighed, all tumour‐bearing mice were euthanised via an intraperitoneal injection of pentobarbital sodium (200 mg/kg) on the 50th day. When these mice revealed the following conditions, we considered terminating this experiment: (1) weight loss over 15%–20% or showing cachexia symptoms; (2) loss of appetite for 5 days or desire to drink for 3 days and (3) unable to stand for 24 h.

### 2.13 Immunohistochemistry

Paraffin‐embedded sections were dewaxed with xylene, hydrated with a graded alcohol series and incubated in H_2_O_2_ for 5 min at room temperature. High‐pressure antigen retrieval was performed with 0.1 mol/L sodium citrate buffer (pH = 6.0). Afterwards, 5% goat serum was used to block the samples for 10 min at room temperature, and primary antibodies diluted to the recommended concentrations with PBS were added. Afterwards, we placed these samples in an incubator and incubated them at 4°C overnight. These tissues were washed with PBS, incubated with secondary antibody at room temperature for 1 h and labelled with horseradish peroxidase. We used DAB for colour development and haematoxylin to restain the nuclei. The sections were stained a blue colour with hydrochloric acid alcohol, dehydrated with a gradient alcohol series, cleared with xylene and sealed with neutral gum. Finally, we observed these samples under a microscope. Negative control samples were generated by using PBS instead of primary antibody in the staining protocol.

### 2.14 Statistical Analysis

SPSS 21.0 and GraphPad Prism 8.0 were used for statistical analysis. The experimental data are expressed as the means ± standard deviations (SDs). One‐way analysis of variance was used for multisample comparisons. The LSD method was used for sample comparisons between each group. *p < 0.05 was considered statistically significant.

## 3 Results

### 3.1 miR‐192 Levels Were Decreased and HOXB9 Levels Were Increased in Malignant Glioma Cells

**FIGURE 1:**
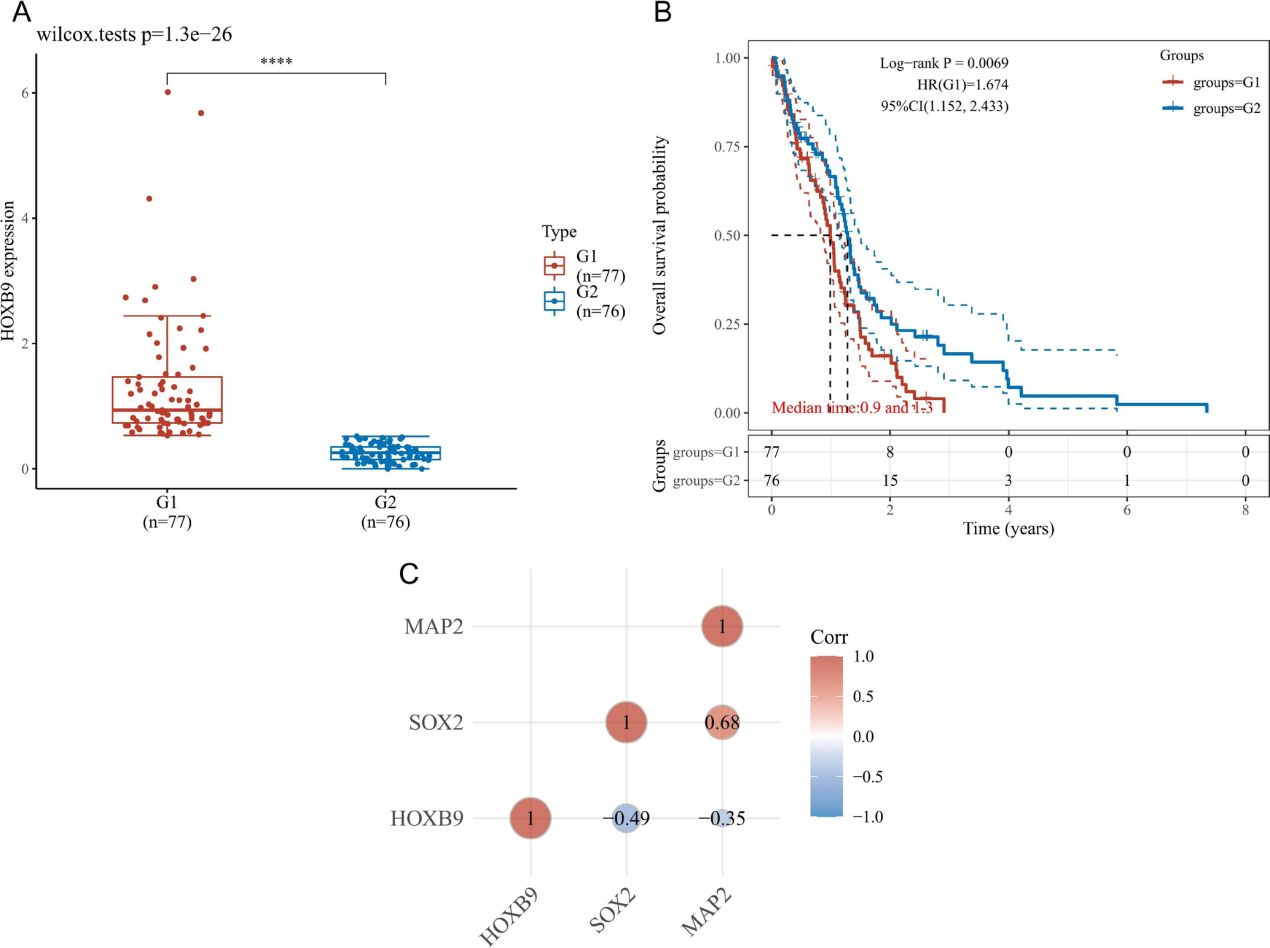
(A) Bioinformatics analysis of miR‐192 pathway‐related factor (HOXB9) expression in glioma. (B) Survival analysis of patients with high and low HOXB9 expression. (C) Correlation analysis between HOXB9 expression and the neural stem cell phenotype.

Initially, we obtained GBM patient data from The Cancer Genome Atlas (TCGA) database and divided the patients into groups according to HOXB9 expression. The results revealed that the prognosis of patients with high HOXB9 expression was poor (*p* < 0.05, Figure 1A,B). Moreover, the expression of HOXB9 and neural stem cell phenotype markers (SOX2/MAP2) were obviously correlated (*p* < 0.05, Figure 1C).

**FIGURE 2:**
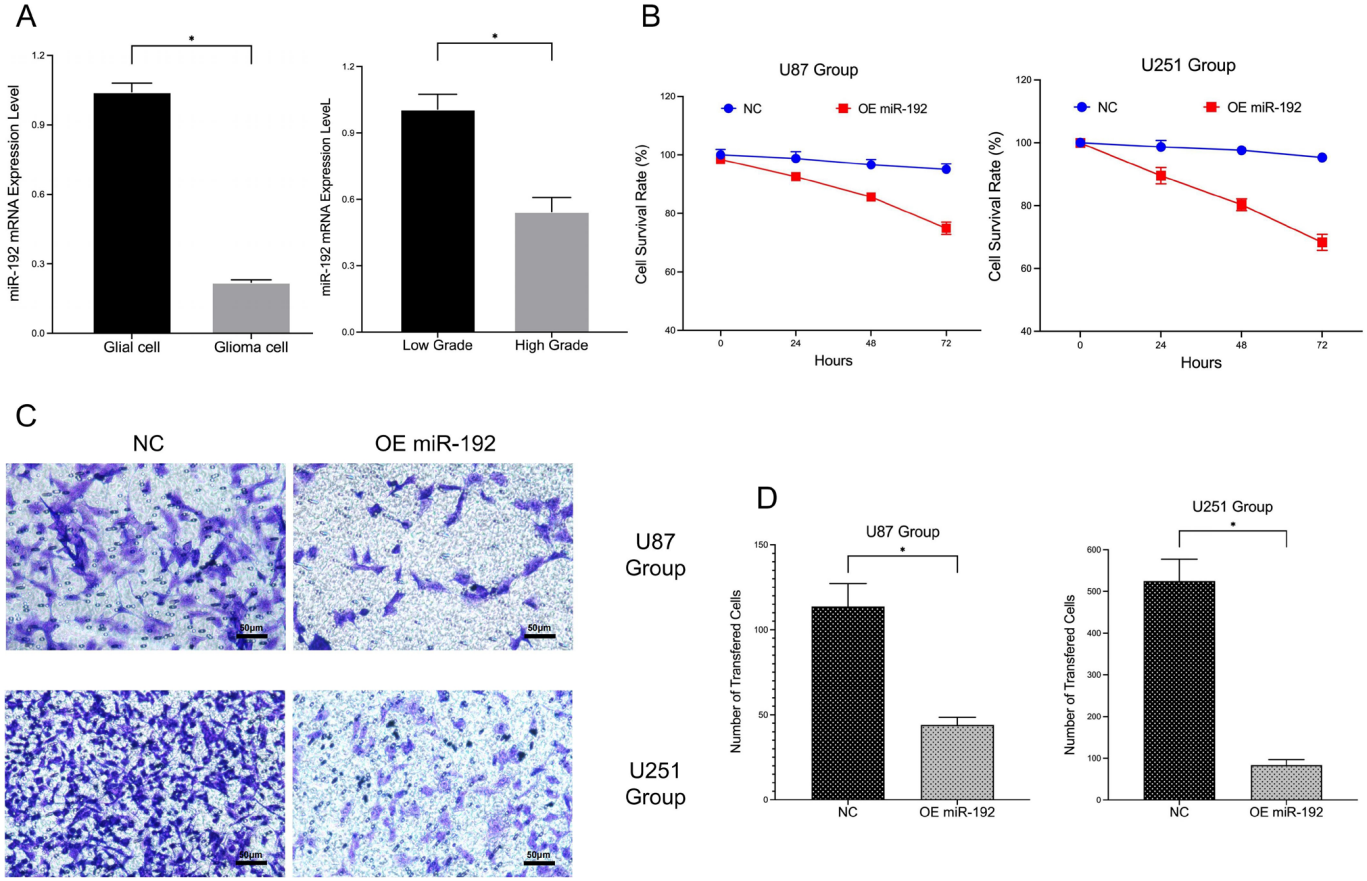
Expression of miR‐192 in glioma samples and its effects on tumour cell proliferation and invasion. (A) PCR detection of miR‐192 expression in cell samples (glial/glioma, low/high grade). (B) CCK‐8 assay using cell models (NC and OE miR‐192). (C) Transwell assay using cell models (NC and OE miR‐192). (D) Statistical data for the different groups (U87 and U251) from the Transwell assay experiment (*p < 0.05, ns *p* > 0.05).

Compared with that in normal glial cells, the content of miR‐192 in glioma cells was obviously lower (0.220 ± 0.010 vs. 1.043 ± 0.021; *p < 0.05). Similarly, compared with that in low‐grade glioma samples (WHO I–II), the level of miR‐192 in high‐grade glioma samples (WHO III–IV) was significantly lower (0.545 ± 0.064 vs. 1.008 ± 0.067; *p < 0.05; Figure 2A).

The CCK‐8 assay revealed that the proliferative ability of glioma cells (U87 and U251) overexpressing miR‐192 was obviously reduced (Figure 2B). Transwell assays revealed that the invasion ability of glioma cells (U87 and U251) overexpressing miR‐192 was significantly reduced (44.0 ± 4.6 vs. 113.7 ± 13.6, 84.0 ± 12.8 vs. 525.0 ± 52.1; *p < 0.05; Figure 2C,D).

These results confirmed that in high‐grade glioma samples, miR‐192 expression was significantly low, and high miR‐192 expression decreased the proliferation and invasion of glioma cells. On this basis, we speculated that miR‐192 may act as an important tumour suppressor in glioma.

### 3.2 miR‐192 Downregulated the Expression of EGR1 and HOXB9 Through Targeted Binding

**FIGURE 3:**
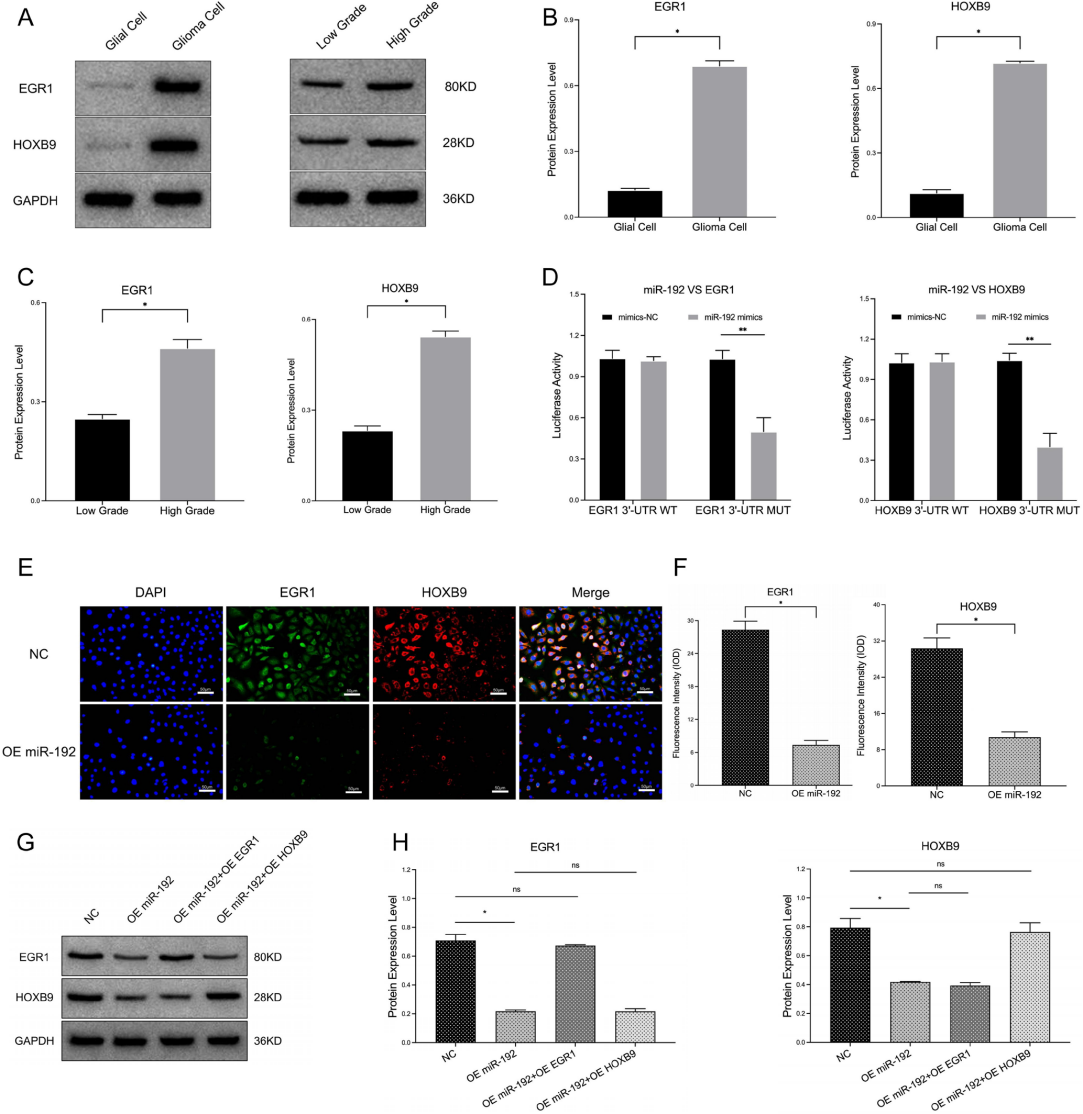
EGR1 and HOXB9 expression and their targeted binding with miR‐192 in glioma samples. (A) WB detection of EGR1 and HOXB9 expression in cell samples (glial/glioma, low/high grade). (B, C) Statistical data of EGR1 and HOXB9 expression in the cell samples. (D) Dual‐luciferase reporter assay of miR‐192 and EGR1 and HOXB9. (E) Cell fluorescence detection of EGR1 and HOXB9 expression in glioma cell lines (NC and OE miR‐192). (F) Statistical data of EGR1 and HOXB9 fluorescence intensity in cell models. (G, H) WB detection of EGR1 and HOXB9 expression in glioma cell models and data statistics (*p < 0.05, ns *p* > 0.05).

WB analysis revealed that, compared with those in normal glial cells, the contents of EGR1 and HOXB9 in glioma cells obviously increased (0.690 ± 0.024 vs. 0.124 ± 0.008, 0.719 ± 0.007 vs. 0.114 ± 0.015; *p < 0.05; Figure 3A,B). Similarly, compared with those in low‐grade glioma samples (WHO I–II), EGR1 and HOXB9 levels were also significantly increased in high‐grade (WHO III–IV) glioma samples (0.463 ± 0.026 vs. 0.249 ± 0.013, 0.543 ± 0.018 vs. 0.233 ± 0.015; *p < 0.05; Figure 3A,C).

Compared with that in the normal control group (mimics NC), the luciferase activity in the experimental group (miR‐192 mimics) with the EGR1 3′‐UTR mutation was obviously lower (0.5 ± 0.1 vs. 1.0 ± 0.0; *p < 0.05). Similarly, the luciferase activity in the experimental group (miR‐192 mimics) with the HOXB9 3′‐UTR was also significantly reduced after mutation (0.4 ± 0.1 vs. 1.0 ± 0.0; *p < 0.05; Figure 3D). Compared with those in normal control cells, the immunofluorescence signals of EGR1 and HOXB9 were obviously lower in glioma cells overexpressing miR‐192 (EGR1: 7.40 ± 0.79 vs. 28.35 ± 1.56; HOXB9: 10.75 ± 1.19 vs. 30.37 ± 2.28; *p < 0.05; Figure 3E,F).

A cell model test revealed that in glioma cells overexpressing miR‐192, the protein levels of EGR1 and HOXB9 were significantly lower (EGR1: 0.217 ± 0.010 vs. 0.710 ± 0.041; HOXB9: 0.419 ± 0.004 vs. 0.794 ± 0.064; * *p* < 0.05). The protein expression of EGR1 clearly increased after simultaneous overexpression of EGR1; thus, there was no significant difference compared with that in the normal control group (0.674 ± 0.006 vs. 0.710 ± 0.041; ns *p* > 0.05). Moreover, the expression of HOXB9 did not obviously differ between the OE miR‐192 + OE EGR1 group and the OE miR‐192 group (0.394 ± 0.020 vs. 0.419 ± 0.004; ns *p* > 0.05). Similarly, the protein content of HOXB9 obviously increased after simultaneous overexpression of HOXB9; thus, there was no significant difference between this group and the normal control group (0.765 ± 0.064 vs. 0.794 ± 0.064; ns *p* > 0.05). Moreover, the expression of EGR1 did not obviously differ between the OE miR‐192 + OE HOXB9 group and the OE miR‐192 group (0.217 ± 0.020 vs. 0.217 ± 0.010; ns *p* > 0.05; Figure 3G,H).

On the basis of these results, we speculate that there is an upstream regulatory relationship between miR‐192 and EGR1/HOXB9. This regulatory factor could inhibit the expression of downstream EGR1 and HOXB9 through targeted binding, thus forming a semi‐open loop that could regulate the malignant phenotypes of glioma. Moreover, there was no interaction between EGR1 and HOXB9.

### 3.3 miR‐192 Inhibited the Malignant Phenotype of Glioma Through the EGR1/HOXB9 Pathway

**FIGURE 4:**
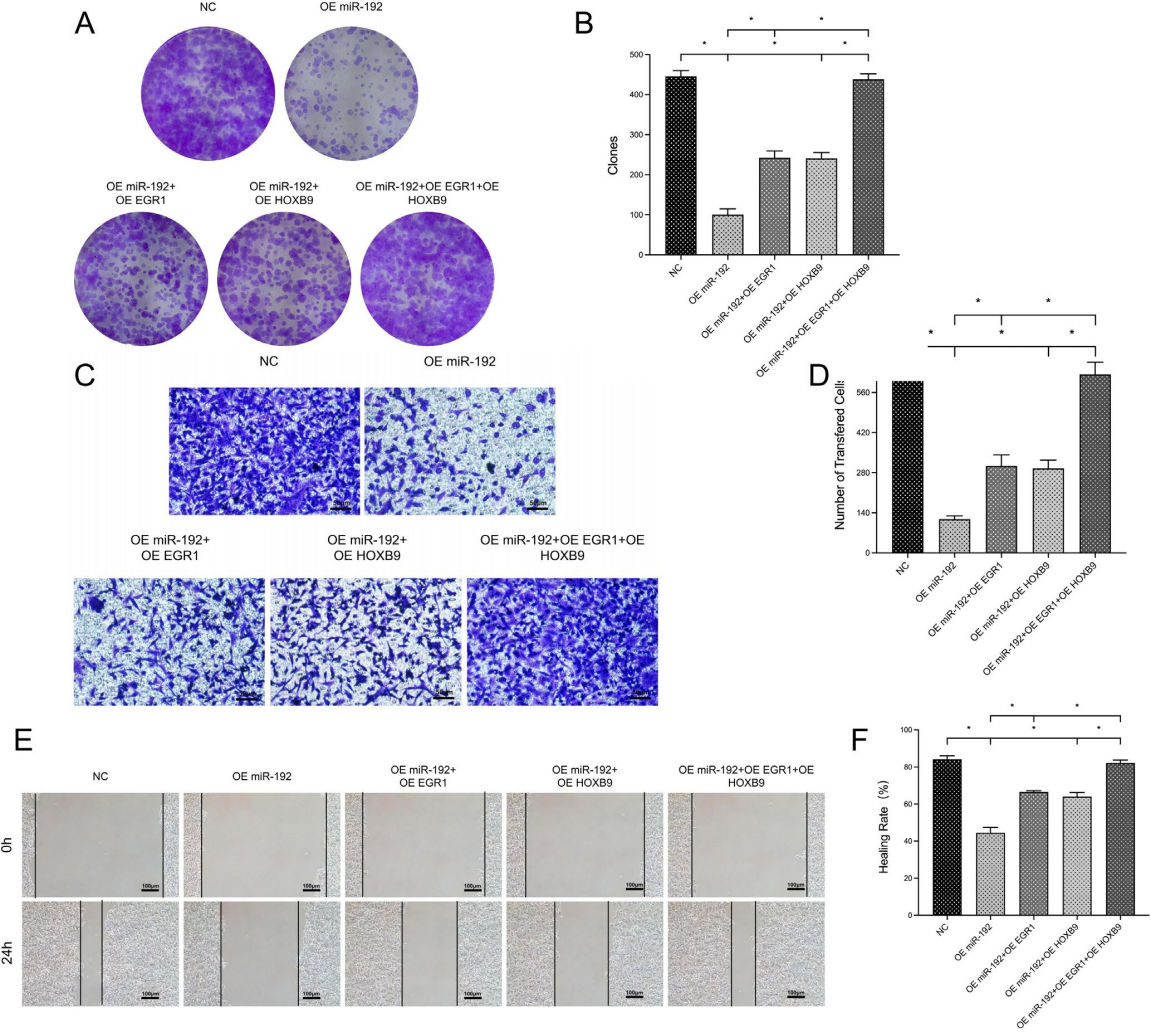
Effects of the EGR1‐HOXB9 loop on the proliferation and invasion in glioma cells. (A, B) Colony formation assay in glioma cell models and data statistics. (C, D) Transwell assay in glioma cell models and data statistics. (E, F) Scratch test in glioma cell models and data statistics (*p < 0.05, ns *p* > 0.05).

Cloning experiments revealed that, compared with those in the normal control group, the number of newborn glioma cells overexpressing miR‐192 was obviously lower (100.3 ± 14.57 vs. 445.7 ± 14.19; *p < 0.05), whereas simultaneous high expression of EGR1 or HOXB9 partially abrogated these changes (242.3 ± 17.01, 240.7 ± 14.57 vs. 100.3 ± 14.57; *p < 0.05). When EGR1 and HOXB9 were overexpressed at the same time, the number of newborn cells essentially returned to normal levels (438.3 ± 13.50 vs. 242.3 ± 17.01, 240.7 ± 14.57; *p < 0.05; Figure 4A,B).

Compared with that in the NC group, the number of migrating cells in the OE miR‐192 group was significantly lower (117.7 ± 11.93 vs. 621.7 ± 26.16; *p < 0.05), while simultaneous expression of EGR1 or HOXB9 partially abrogated these changes (303.7 ± 38.59, 295.0 ± 29.21 vs. 117.7 ± 11.93; *p < 0.05). When we overexpressed EGR1 and HOXB9 together, the number of migrating cells returned to normal levels (623.0 ± 42.67 vs. 303.7 ± 38.59, 295.0 ± 29.21; *p < 0.05; Figure 4C,D).

Scratch tests revealed that, compared with those in the NC group, the proportion of migrating cells in the OE miR‐192 group was obviously lower (44.49 ± 2.96 vs. 84.15 ± 1.86; *p < 0.05), while simultaneous overexpression of EGR1 or HOXB9 partially abrogated these changes (66.53 ± 0.71, 64.01 ± 2.27 vs. 44.49 ± 2.96; *p < 0.05). When we overexpressed EGR1 and HOXB9 together, the proportion of migrating cells essentially returned to normal levels (82.18 ± 1.53 vs. 66.53 ± 0.71, 64.01 ± 2.27; *p < 0.05; Figure 4E,F).

On the basis of these experimental data, we speculated that miR‐192 could regulate the malignant phenotype of glioma cells through the EGR1‐HOXB9 loop, thereby inhibiting tumour cell proliferation, invasion and migration.

### 3.4 miR‐192 Inhibited Glioma Stem Transformation Through EGR1/HOXB9

Immunofluorescence detection revealed that in glioma cells overexpressing miR‐192, the mesenchymal transition marker (N‐cad) was significantly downregulated (1.316 ± 0.341 vs. 18.35 ± 1.217; *p < 0.05). The content of this protein correspondingly increased after simultaneous overexpression of EGR1 or HOXB9 (6.481 ± 0.702, 6.664 ± 0.678 vs. 1.316 ± 0.341; *p < 0.05). When EGR1 and HOXB9 were overexpressed at the same time, the N‐cad content returned to normal levels (18.08 ± 2.246 vs. 6.481 ± 0.702, 6.664 ± 0.678; *p < 0.05).

**FIGURE 5:**
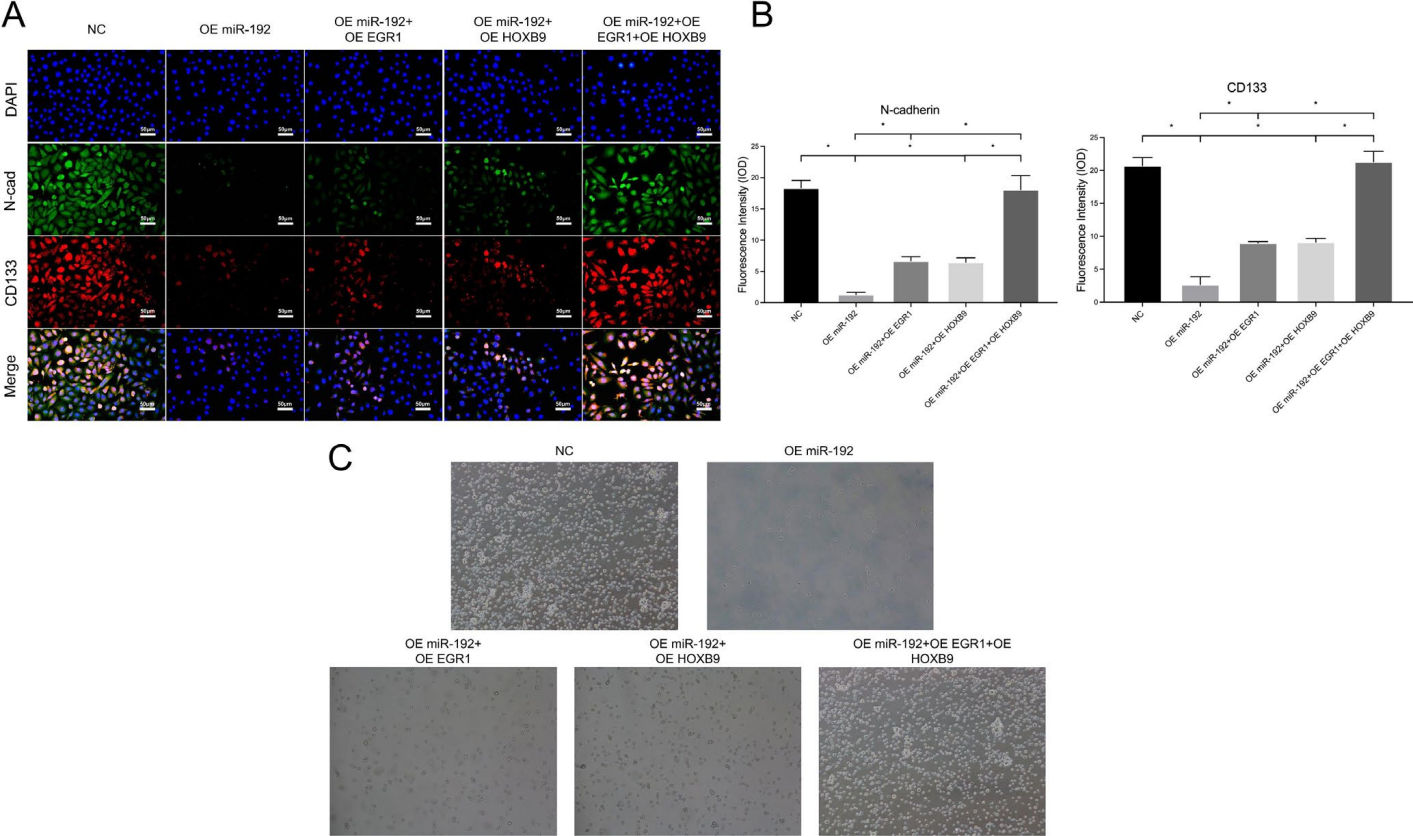
Effects of the EGR1‐HOXB9 loop on the migration of glioma cells and its regulation of tumour cell stemness. (A, B) MT marker (N‐cad)/stemness marker (CD133) detection in glioma cell models and data statistics. (C) Observation of stem cell spheres in glioma cell models (*p < 0.05, ns *p* > 0.05).

Similarly, in glioma cells overexpressing miR‐192, the expression of a stem cell marker (CD133) was obviously downregulated (2.653 ± 1.197 vs. 20.73 ± 1.235; *p < 0.05), whereas simultaneous high expression of EGR1 or HOXB9 partially increased the CD133 content (8.978 ± 0.215, 9.119 ± 0.578 vs. 2.653 ± 1.197; *p < 0.05). If we overexpressed EGR1 and HOXB9 together, CD133 expression returned to normal levels (21.32 ± 1.577 vs. 8.978 ± 0.215, 9.119 ± 0.578; *p < 0.05; Figure 5A,B).

Morphological observations revealed that the number of GSCs that formed stem spheres significantly decreased after the overexpression of miR‐192, whereas the number of GSC stem spheres correspondingly increased with the simultaneous high expression of EGR1 or HOXB9. When we overexpressed EGR1 and HOXB9 at the same time, the number of GSC stem spheres essentially returned to normal levels (Figure 5C).

On the basis of these results, we speculated that miR‐192 could promote MT in glioma cells through the EGR1‐HOXB9 loop, thus regulating their stemness.

### 3.5 miR‐192 Blocked Glioma Cell Tumorigenesis In Vivo Through the EGR1‐HOXB9 Loop

**FIGURE 6:**
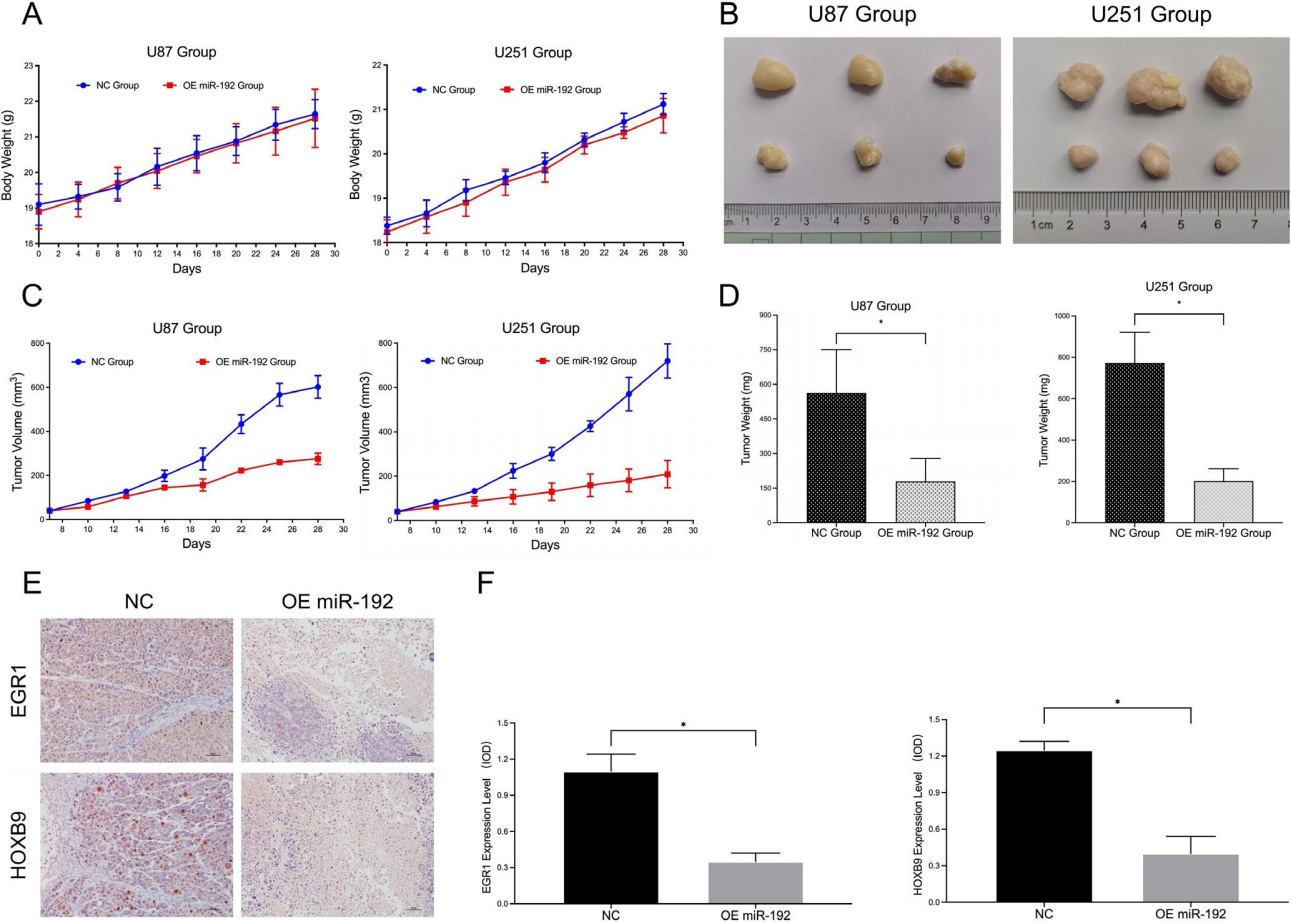
Effects of the EGR1‐HOXB9 loop on the tumourigenesis of glioma cells in vivo. (A) Growth curves of tumour‐bearing mice in different groups (NC and OE miR‐192). (B) In vitro tumour samples from tumour‐bearing mice in different groups (NC and OE miR‐192). (C) In vivo measurement of tumour volume in tumour‐bearing mice in different groups (NC and OE miR‐192). (D) In vitro tumour weight detection and statistical analysis. (E) IHC staining of tumour samples in vitro. (E, F) IOD detection of IHC‐stained glioma samples (*p < 0.05, ns *p* > 0.05).

We selected glioma cells (U87 and U251) in the logarithmic growth phase and constructed xenograft models through subcutaneous inoculation. Growth curve measurements under in vivo conditions revealed no significant difference in body weight between the NC and OE miR‐192 groups, indicating that the growth status of the transplanted models in the different groups was consistent (Figure 6A).

In vivo tumour volume detection revealed that, compared with that in the NC group, the tumour volume in the OE miR‐192 group clearly decreased beginning on the 16th day (Figure 6C). Compared with that in the NC group, the tumour weight in the OE miR‐192 group was significantly lower (U87 group: 179.0 ± 99.46 vs. 563.0 ± 187.0; U251 group: 202.0 ± 58.80 vs. 773.0 ± 148.2; *p < 0.05; Figure 6B,D).

Immunohistochemical staining of isolated glioma samples revealed that, compared with those in the NC group, EGR1 and HOXB9 protein levels in glioma samples from the OE miR‐192 group were obviously lower (U87 group: 0.35 ± 0.07 vs. 1.10 ± 0.14; U251 group: 0.40 ± 0.14 vs. 1.25 ± 0.07; *p < 0.05; Figure 6E,F). These findings confirmed that miR‐192 could also inhibit the expression of EGR1 and HOXB9 through targeted binding in vivo.

On the basis of these results, we speculated that in vivo, miR‐192 could also inhibit the proliferation and tumorigenesis of glioma cells through the EGR1–HOXB9 loop.

## 4 Discussion

### 4.1 MT in Glioma Cells

MT is considered an important way for malignant tumour cells to acquire the ability to invade and migrate; at the same time, this factor may be a key step in tumour metastasis. Previous studies revealed that this process is usually accompanied by changes in a variety of mesenchymal molecular markers. When MT occurs, the expression of E‐cadherin is usually downregulated, whereas N‐cadherin, vimentin and β‐catenin expression is upregulated. The role of MT in epithelial tumours, such as breast, colorectal, pancreatic, thyroid and lung cancer, has been extensively studied [12, 13].

However, whether MT also occurs in high‐grade glioma has been controversial; thus, few relevant studies have focused on this topic. Some researchers believe that E‐cadherin downregulation usually occurs in aggressive glioma cells and that its expression level is negatively correlated with tumour cell invasion and migration. Camand demonstrated that N‐cadherin was abnormally expressed in human glioma samples, which promoted tumour cell malignant phenotypes. Although relevant studies have suggested that the expression of E‐cadherin and N‐cadherin during MT in glioma cells is not completely consistent with that in other tumours of epithelial origin, some scholars believe that MT is also related to the increased proliferation and invasion of glioma cells in high‐grade glioma samples. Some studies have revealed a certain correlation between interstitial changes in GBM and their progressive malignant phenotype. A study by Tso demonstrated that several genes expressed in interstitial tissues were overexpressed in GBM samples, which indicated that this tumour could have high interstitial and invasive properties [14, 15].

These findings indicate that ‘cadherin switching’ is not necessary during the MT process in glioma and that these cells may not experience typical ‘MT switching’. Research has confirmed that MT is a multistep cellular reprogramming process regulated by complex molecular networks. Many factors, such as MT‐inducing transcription factors, cell growth factors, the TGF‐β signalling pathway, hypoxia, ncRNAs and epigenetic factors, could participate in this progression. Thus, some scholars believe that mechanisms other than ‘cadherin switching’ may play important roles in the regulation of MT in glioma. Therefore, we believe that MT in glioma cannot be detected only by researching classic ‘cadherin switching’ markers; therefore, we investigated other possible mechanisms [16, 17].

Our study confirmed that MT also occurs in glioma cells, and this process is often accompanied by abnormal expression of a specific marker (N‐cadherin). On the basis of these results, we believe that MT marker expression is correlated with malignant phenotypes in glioma.

### 4.2 GSCs and MT

Relevant studies have shown that self‐renewal is an important characteristic of stem cells and that CSCs can form tumour spheres in serum‐free and suspension culture conditions. CSCs account for a small proportion of tumour cells; these cells have the ability to differentiate in multiple directions and form tumours in vivo. Some researchers believe that CSCs play a decisive role in tumour initiation and progression, which may be the basic reason for tumour invasion, recurrence and therapy resistance. Some scholars believe that there is an inseparable relationship between MT and CSCs in tumours of epithelial origin. Mani reported that breast cancer cells that underwent MT usually exhibited stem‐like properties. To date, this discovery has been confirmed in various tumours, such as lung, colorectal and kidney cancer [18, 19].

However, whether the relationship between MT and GSCs has been fully studied is not clear. Since gliomas originate from glial cells, Mahabir proposed that glial‐to‐mesenchymal transition (GEMT) could occur in glioma cells in place of traditional MT, but this proposal has not been widely adopted. In recent years, studies have confirmed that, similar to epithelial‐derived CSCs, GSCs can also undergo MT under certain conditions. Relevant studies have shown that the number of glial spheres in second‐generation U87 cells that underwent MT is significantly greater than that in control cells. Thus, glioma cells that undergo MT could exhibit stem‐like characteristics [20, 21].

The results of this study revealed that the miR‐192‐EGR1/HOXB9 loop might be involved in the regulation of glioma interstitial transformation, which was consistent with changes in glioma cell stemness. On the basis of these results, we believe that this target signalling pathway could promote MT and ultimately induce a stemness phenotype in glioma cells.

### 4.3 GSCs and High‐Grade Gliomas

Although treatments such as surgery, radiotherapy and chemotherapy can eliminate most proliferating tumour cells, they still cannot cure cancer completely. Therefore, some scholars have proposed that stem cells might be intrinsic factors that maintain the continuing growth of tumours during their origin and evolution. These hypotheses suggest that CSCs might enable tumour cells to possess characteristics such as proliferation, infiltration, and metastasis. Therefore, reducing the stemness of tumour cells may be a new strategy for the treatment of these patients. At present, GSCs are identified through molecular markers, including CD133, Nestin and CD15. Clinical data have shown that CD133 expression is closely related to the tumour grade and clinical prognosis of these patients. Lv reported that the expression of Nestin in high‐grade gliomas was significantly greater than that in low‐grade gliomas [22].

GSCs are a rare cell subpopulation characterised by self‐renewal and tumourigenicity. The proliferation of GSCs is closely related to increased resistance to drugs, radiation and cellular stress in glioma. Research has shown that these cells can differentiate into mesenchymal subtypes, and MT is closely related to this process. Previous studies revealed that TGF‐β1 could induce MT and enhance the stemness of glioma cells/GSCs during tumour metastasis, which suggested that TGF‐β1 could be used as a molecular target for the clinical treatment of these patients [23].

The results of our study revealed that changes in malignant phenotypes were positively correlated with MT marker expression in glioma cells. Therefore, we believe that MT might play important roles in the regulation of the malignant phenotypes of glioma cells.

### 4.4 Effect of the miR‐192‐EGR1/HOXB9 Loop on the Malignant Phenotypes of Glioma Cells

#### 4.4.1 Regulatory Role of the miR‐192‐EGR1/HOXB9 Loop in Malignancy

1. It has been confirmed that miR‐192 plays key roles in a variety of cancers. The expression of this factor is usually downregulated in liver, gastric and lung cancers. It can inhibit the proliferation and invasion of tumour cells, which suggests that this factor may have the potential to be a tumour suppressor [24].
2. EGR1 is a transcription factor that has been confirmed to play important roles in the pathogenesis of various cancers. This factor affects a variety of genes related to cell growth, differentiation and apoptosis. For example, in prostate and bone cancer, EGR1 is upregulated, which may aggravate tumour progression by promoting the proliferation and inhibiting the apoptosis of tumour cells [9].
3. HOXB9 is a member of the homeobox gene family, which is involved in embryonic development and cell differentiation by encoding transcription factors. In recent years, HOXB9 has been shown to be upregulated in a variety of cancers (such as lung, breast and colon cancers) and ultimately promotes cancer progression by mediating cell proliferation, migration and invasion. Recently, the role of the miR‐192‐EGR1/HOXB9 loop in the pathogenesis of tumours has attracted increasing attention from academic researchers.
4. Related studies have shown that in pancreatic cancer, the expression of miR‐192 is obviously downregulated, whereas EGR1 and HOXB9 are upregulated, and these factors may be involved in the regulation of malignant phenotypes in tumour cells [11].

Our research demonstrated that miR‐192 could downregulate the expression of EGR1 and HOXB9 via targeted binding. Therefore, inhibiting the proliferation, invasion and migration of glioma cells simultaneously inhibits tumour cell tumourigenesis in vivo. These results suggest that miR‐192 could act as a tumour suppressor in glioma and that this factor could inhibit the malignant phenotypes of glioma cells via the EGR1–HOXB9 loop.

#### 4.4.2 HOXB9 Regulates the Stemness of Glioma Cells

Research has shown that HOXB9 might play important roles in regulating MT in tumour cells and the self‐renewal ability of stem cells, thus affecting the malignant phenotypes of tumour cells.

First, a large amount of experimental data confirmed that HOXB9 overexpression could promote MT in various tumours (such as breast, lung and pancreatic cancers). These specific manifestations include the upregulation of mesenchymal cell markers as well as changes in cell morphology and increased tumour cell invasion and migration. Interference with HOXB9 abrogated these effects, indicating that HOXB9 plays key roles in promoting MT in tumour cells.

Second, GSCs are generally considered to have unlimited self‐renewal capacity and the potential for multidirectional differentiation. These characteristics enable them to play important roles in tumour growth and invasion in glioma. The overexpression of HOXB9 could increase the stemness of glioma cells, thus increasing chemotherapy resistance in these cases. Interference with HOXB9 could inhibit the stem properties of glioma cells. These results indicate that HOXB9 plays key roles in regulating the stemness of these cells [10].

Our research revealed that both EGR1 and HOXB9 promote MT in glioma cells. As an upstream regulator, miR‐192 can block MT progression through the EGR1‐HOXB9 loop in glioma samples and ultimately reduce the stemness of these cells. On the basis of these results, we believe that miR‐192 blocks MT in glioma cells and inhibits their stemness via the EGR1‐HOXB9 loop, which ultimately regulates the malignant phenotypes of these tumour cells.

## 5 Conclusion and Prospects

Our study demonstrated that miR‐192 was significantly downregulated in glioma tissues and that this factor could inhibit MT in glioma cells through the EGR1‐HOXB9 loop while reducing their stemness and ultimately inhibiting their malignant phenotypes. However, the molecular mechanisms underlying the interactions between the key factors in this regulatory loop and their target cells and the ability of these factors to regulate glioma cell stemness need to be further studied in subsequent cellular and molecular research. Moreover, the effects of this regulatory loop on malignant progression, recurrence, radiotherapy/chemotherapy tolerance and immunotherapy resistance in glioma patients have not been elucidated. This area needs to be clarified through subsequent cellular/molecular/animal/clinical trials. On the basis of these results, we believe that miR‐192 could act as a tumour suppressor in glioma and that its related downstream pathway could be a new therapeutic target for these patients.

## Author Contributions

**Guo‐Wei Li:** conceptualization (lead), formal analysis (lead), funding acquisition (equal), methodology (lead), project administration (equal), resources (equal), software (lead), validation (lead), visualization (lead), writing – original draft (lead). **Yan‐Ping Jin:** data curation (lead), formal analysis (supporting), funding acquisition (equal), investigation (lead), methodology (supporting), supervision (lead), validation (supporting), visualization (supporting), writing – review and editing (lead). **Min‐Feng Sheng:** project administration (equal), resources (equal), supervision (equal), visualization (equal), writing – review and editing (supporting).

## Ethics Statement

This study was approved by the Ethics Committee of Nanjing Normal University of Special Education and was performed in accordance with ethical standards in the Declaration of Helsinki. We confirm that this study was performed according to institutional rules for animal experiments and biodiversity rights.

## Consent

The authors have nothing to report.

## Conflicts of Interest

The authors declare no conflicts of interest.

## Data Availability

The datasets analyzed in this study are available from the corresponding author upon reasonable request.
